# Data set concerning the use of social networking sites and mental health problems among the young generation in Bangladesh

**DOI:** 10.1016/j.dib.2021.107593

**Published:** 2021-11-19

**Authors:** Md. Rabiul Islam, Md. Ismail Tushar, Sanjida Jannath, Amena Ahmed Moona, Shahinur Akter, Sardar Mohammad Ashraful Islam

**Affiliations:** a Department of Pharmacy, University of Asia Pacific, 74/A Green Road, Farmgate, Dhaka 1205, Bangladesh; b Department of Pharmaceutical Sciences, North South University, Bashundhara, Dhaka 1229, Bangladesh

**Keywords:** Social networking sites, Facebook, Mental health, Loneliness, Depression, Anxiety, Sleep disturbance

## Abstract

The article depicts a unique dataset of responses from 791 adults to a self-made questionnaire of five sections sent via Google survey tool (Google form) from February 4, 2021, to March 18, 2021 [1]. We collected responses for establishing a paradigm of the relationship between the social networking sites (SNS) use and four dimensions of psychological distress including depression, anxiety, loneliness, and sleep disturbances. Facebook is the most popular social media in Bangladesh, we observed 669 Facebook users and 122 non-Facebook-users aged between 15 to 40 years in this data set. We analyzed the collected data using the Microsoft Excel (version 2016) and presented as frequencies and percentages based on responses to the whole survey. The survey contained items focusing on (i) sociodemographic information, (ii) usage patterns of SNS, (iii) assessment of mental health problems. We collected responses from all across the country regardless of sociodemographic background. Therefore, government authorities and healthcare providers can use this data for dealing with the mental health issues concerning the use of SNS.

## Specifications Table

**Table:** 

Subject	Social science
Specific subject area	Social media, Psychology
Type of data	Table and figure
How data were acquired	Google survey tools (Google Forms)
Data format	Raw and analysed
Parameters for data collection	Respondents were chosen based on convenient sampling technique. We collected responses from participants aged between 15–40 years who were of Bangladeshi ethnicity and living in Bangladesh. Inclusion criteria were social media users who were willing to participate in this study irrespective of background or socio-demographic variables.
Description of data collection	We conducted this concurrent cross-sectional study from February 4, 2021, to March 18, 2021 using Google survey tools (Google Forms). A self-reported questionnaire was sent to the participants through e-mail, Facebook, Messenger, WhatsApp, Instagram, etc. The structured questionnaire was designed to collect the general information about the participants. We applied different scales (UCLA-8, PHQ-9, GAD-7, and PSQI) for psychometric measurements. The survey questionnaire and all the answers to the questions in English have been provided as supplementary files 1 and 2.
Data source location	Researchers from University of Asia Pacific, Dhaka, have collected data from across the Bangladesh.
Data accessibility	Data is within this article
Related research article	M.R. Islam, S. Jannath, A.A. Moona, S. Akter, M.J. Hossain, S.M.A. Islam. Association between the use of social networking sites and mental health of young generation in Bangladesh: A cross-sectional study, J. Community Psychol. 49(7) (2021) 2276–2297. https://doi.org/10.1002/jcop.22675

## Value of the Data

• This data set contains responses from people of a target age limit (15–40) who belong to the young generation. The data set shows the vulnerability of mental health of the young generation in Bangladesh due to the use of SNS.
• The data can help researchers finding out the factors for poor mental health concerning the use of SNS among the young Bangladeshi population.
• Government authorities and non-government organizations can use this data set as part of their policymaking and developing models to improve mental health related to the use of social media.
• This evidence-based study can utilize in designing prevention programs for mental health issues like loneliness, anxiety, depression, and sleep disturbances by policymakers.
• The data set can provide in-depth insights into the impacts of social media in our lives by causing mental health problems.

## 1 Data Description

Social media has transformed our way of communication and interaction with people. It explicitly holds a major contributing factor for killing time. Is it good or bad in our day-to-day life - is a big question for us. It is rational to conduct studies on its relation to our mental health condition. As an attempt, this cross-sectional study was planned, designed, and carried out. We constructed the survey questionnaire in separate sections to measure four mental illnesses by following internationally validated scales: the UCLA Loneliness Scale-8 (UCLA-8), Patient Health Questionnaire-9 (PHQ-9), 7-item Generalized Anxiety Disorder (GAD-7) Scale, and Pittsburgh Sleep Quality Index (PSQI) [2], [3], [4], [5].

**Fig. 1:**
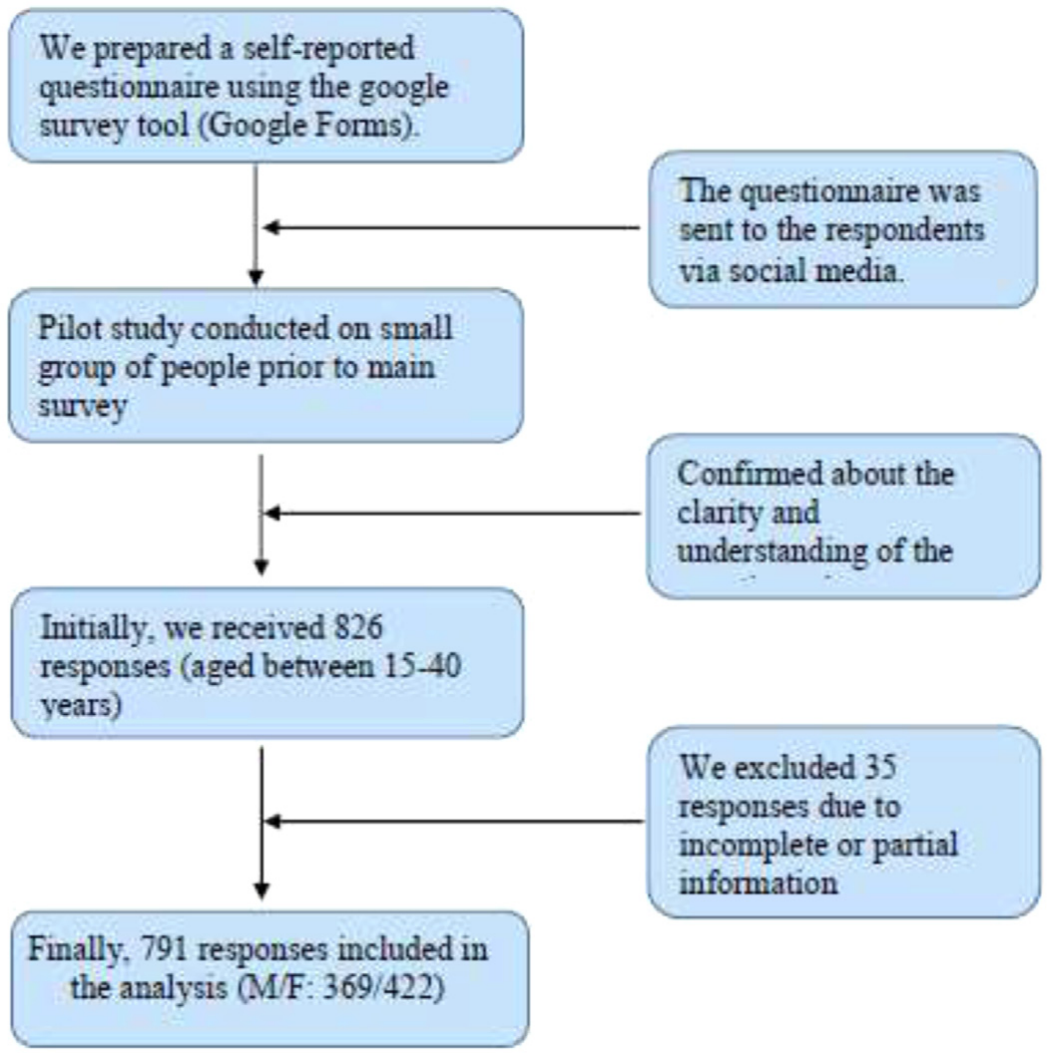
Flowchart of collecting responses from the participants.

The survey data set provides insights about the usage pattern and triggering factors for mental health problems due to the use of SNS. It also provides perceptions of what people think about social media and SNS-induced depression, anxiety, loneliness, and sleep disorders. We obtained responses from authentic users of a specified age limit and different sociodemographic backgrounds in Bangladesh. The data set comprises (i) assessment of loneliness (UCLA-8) in Table 1, (ii) Assessment of depression (PHQ-9) in Table 2, (iii) Assessment of anxiety (GAD-7) in Table 3, (iv) Assessment of sleep disturbances (PSQI) in Table 4. It also presents a flowchart of the collection and exclusion procedure of data (viii) Fig. 1. Based on the present dataset, it is difficult to conclude whether the mental health of the young Bangladeshi population is affected by the use of SNS or the COVID-19 pandemic. Moreover, the COVID-19 pandemic and its responses have enormously impacted individuals’ mental health, social life, physical health, etc., in Bangladesh [6], [7], [8], [9], [10], [11], [12], [13], [14]. People were heavily involved with SNS than ever due to the ongoing COVID-19 responses. Therefore, frequent use of SNS during the COVID-19 period might create additional mental health problems.

**Table 1:** Distribution of responses based on the UCLA Loneliness Scale-8 (UCLA-8).

How often the respondents feel the below statements descriptive of you in the past 30 days?	Frequency (n)	Percentage (%)
I lack companionship		
I never feel this way	213	26.93
I rarely feel this way	220	27.81
I sometimes feel this way	267	33.76
I often feel this way	91	11.50
There is no one I can turn to		
I never feel this way	286	36.16
I rarely feel this way	214	27.05
I sometimes feel this way	217	27.43
I often feel this way	74	9.36
I am an outgoing person		
I never feel this way	180	22.76
I rarely feel this way	164	20.73
I sometimes feel this way	269	34.01
I often feel this way	178	22.50
I feel left out		
I never feel this way	264	33.38
I rarely feel this way	189	23.89
I sometimes feel this way	253	31.98
I often feel this way	85	10.75
I feel isolated from others		
I never feel this way	259	32.74
I rarely feel this way	176	22.25
I sometimes feel this way	251	31.73
I often feel this way	105	13.28
I can find companionship when I want it		
I never feel this way	138	17.45
I rarely feel this way	176	22.25
I sometimes feel this way	268	33.88
I often feel this way	209	26.42
I am unhappy being so withdrawn		
I never feel this way	244	30.85
I rarely feel this way	194	24.53
I sometimes feel this way	265	33.50
I often feel this way	88	11.12
People are around me but not with me		
I never feel this way	212	26.80
I rarely feel this way	187	23.64
I sometimes feel this way	271	34.26
I often feel this way	121	15.30

**Table 2:** Distribution of responses based on the Patient Health Questionnaire-9 (PHQ-9).

How often the respondents bothered by any of the below problems since last 2 weeks?		Frequency (n)		Percentage (%)
Little interest or pleasure in doing things				
Not at all		208		26.30
Several days		375		47.41
More than half of the days		115		14.54
Nearly everyday		93		11.75
Feeling down, depressed, or hopeless				
Not at all		257		32.49
Several days		334		42.23
More than half of the days		86		10.87
Nearly everyday		114		14.41
Trouble falling or staying asleep, or sleeping too much				
Not at all		286		36.16
Several days		296		37.42
More than half of the days		99		12.52
Nearly everyday		110		13.90
Feeling tired or having little energy				
Not at all		207		26.17
Several days		346		43.74
More than half of the days		109		13.78
Nearly everyday		129		16.31
Poor appetite or overeating				
Not at all		300		37.93
Several days		297		37.55
More than half of the days		107		13.53
Nearly everyday		87		10.99
Feeling bad about yourself or that you are a failure or have let yourself down				
Not at all		359		45.39
Several days		234		29.58
More than half of the days		79		9.99
Nearly everyday		119		15.04
Trouble concentrating on things				
Not at all		337		42.61
Several days		231		29.20
More than half of the days		83		10.49
Nearly everyday		140		17.70
Moving or speaking so slowly that other people could have noticed? Or the opposite				
Not at all		392		49.56
Several days		268		33.88
More than half of the days		73		9.23
Nearly everyday		58		7.33
Thoughts that you would be better off dead or of hurting yourself in some way				
Not at all		472		59.67
Several days		198		25.03
More than half of the days		58		7.33
Nearly everyday		63		7.97

**Table 3:** Distribution of responses based on the 7-item Generalized Anxiety Disorder (GAD-7) Scale.

How often the respondents bothered by the following problems in last two weeks?	Frequency (n)	Percentage (%)
Feeling nervous, anxious, or on edge		
Not at all	291	36.78
Several days	328	41.47
More than half of the days	82	10.37
Nearly everyday	90	11.38
Not being able to stop or control worrying		
Not at all	270	34.13
Several days	289	36.54
More than half of the days	104	13.15
Nearly everyday	128	16.18
Worrying too much about different things		
Not at all	245	30.97
Several days	289	36.54
More than half of the days	111	14.03
Nearly everyday	146	18.46
Felt trouble in relaxing		
Not at all	295	37.29
Several days	289	36.54
More than half of the days	85	10.75
Nearly everyday	122	15.42
Being so restless that it's hard to sit still		
Not at all	352	44.50
Several days	258	32.62
More than half of the days	97	12.26
Nearly everyday	84	10.62
Becoming easily annoyed or irritable		
Not at all	266	33.63
Several days	294	37.17
More than half of the days	86	10.87
Nearly everyday	145	18.33
Feeling afraid as if something awful might happen		
Not at all	331	41.85
Several days	262	33.12
More than half of the days	68	8.60
Nearly everyday	130	16.43

**Table 4:** Distribution of responses based on the Pittsburgh Sleep Quality Index (PSQI).

Sleep quality measurement parameters during last month	Frequency (n)	Percentage (%)
When you have usually gone to bed at night?		
8.00 PM to 10.00 PM	47	5.94
10.01 PM to 12.00 AM	405	51.20
12.01 AM to 2.00 AM	232	29.33
2.01 AM to 5.00 AM	107	13.53
How long (in minutes) has it usually takes you to fall asleep each night?		
Within 15 min	322	40.71
16–30 min	281	35.52
31–60 min	95	12.01
More than 60 min	93	11.76
When have you usually gotten up in the morning?		
Within 5.00 AM	61	7.71
5.01 AM to 7.00 AM	286	36.16
7.01 AM to 9.00 AM	245	30.97
After 9.00 AM	199	25.16
How many hours of actual sleep did you get at night?		
Less than 4 h	64	8.09
4 to 6 h	440	55.63
7 to 8 h	254	32.11
More than 8 h	33	4.17
How many hours you spend in bed?		
Less than 5 h	13	1.64
5 to 7 h	389	49.18
8 to 10 h	340	42.99
More than 10 h	49	6.19
Trouble sleeping because you cannot get to sleep within 30 min		
Not during last month	386	48.80
Less than once a week	177	22.38
Once or twice a week	102	12.89
Three or more in week	126	15.93
You wake up in the middle of night or early in the morning		
Not during last month	294	37.17
Less than once a week	226	28.57
Once or twice a week	142	17.95
Three or more in week	129	16.31
You have to get up to use the bathroom		
Not during last month	304	38.43
Less than once a week	208	26.30
Once or twice a week	153	19.34
Three or more in week	126	15.93
Trouble in sleep because you cannot breathe comfortably		
Not during last month	466	58.91
Less than once a week	177	22.38
Once or twice a week	92	11.63
Three or more in week	56	7.08
Trouble in sleep because of cough or snore loudly		
Not during last month	427	53.98
Less than once a week	195	24.65
Once or twice a week	79	9.99
Three or more in week	90	11.38
Trouble in sleep because of feeling too cold		
Not during last month	362	45.76
Less than once a week	208	26.30
Once or twice a week	139	17.57
Three or more in week	82	10.37
Trouble in sleep because of feeling too hot		
Not during last month	414	52.33
Less than once a week	180	22.76
Once or twice a week	113	14.29
Three or more in week	84	10.62
You had bad dreams		
Not during last month	338	42.73
Less than once a week	254	32.11
Once or twice a week	138	17.45
Three or more in week	61	7.71
You have pain during sleep		
Not during last month	492	62.20
Less than once a week	157	19.85
Once or twice a week	93	11.76
Three or more in week	49	6.19
Trouble in sleep because of other reasons		
Not during last month	352	44.50
Less than once a week	203	25.67
Once or twice a week	144	18.20
Three or more in week	92	11.63
How often have you taken medicines to help you sleep?		
Not during last month	584	73.83
Less than once a week	99	12.52
Once or twice a week	59	7.46
Three or more in week	49	6.19
How often have you had trouble staying awake while driving, eating meals, etc.?		
Not during last month	384	48.55
Less than once a week	221	27.94
Once or twice a week	116	14.66
Three or more in week	70	8.85
How many times you face problems to maintain program or other important case?		
Not during last month	383	48.42
Less than once a week	217	27.43
Once or twice a week	127	16.06
Three or more in week	64	8.09
How would you rate your sleep quality overall?		
Very good	218	27.56
Fairly good	376	47.53
Fairly bad	111	14.04
Very bad	86	10.87

## 2 Experimental Design, Materials and Methods

It was not feasible to carry on a face-to-face population-based study due to the ongoing COVID-19 pandemic. Therefore, we designed a self-administered questionnaire using google survey tools (Google Forms) and sent it to the participants through various means like Facebook messenger, email, Instagram, WhatsApp, etc. Inclusion criteria were: any Bangladeshi within 15–40 years who has a social media account or SNS user. Initially, we received 826 responses from February 4, 2021, to March 18, 2021. After careful evaluation of dada, we discarded 35 responses due to the partial or incomplete information. We involved people from different education levels, economic statuses, and occupations in this study. Also, we kept the required option for each question in the Google Form. The survey questionnaire contained five sections. The first section was regarding the socio-demographic profiles and the usage pattern of social media in the respondents. Seven questions regarding socio-demographic profile followed by names of social media use, time spent, number of friends and groups, what they think of social media affecting their mental health, etc. questions were involved.

The second section had eight questions about “how often the respondents feel the below statements descriptive of you in the past 30 days?” to figure out loneliness. Each question had four options: I never feel this way, I rarely feel this way, I sometimes feel this way, I often feel this way. The third section had nine questions about “how often the respondents bothered by any of the below problems since last two weeks?” with four options: not at all, several days, more than half of the days, nearly every day to figure out depression among the participants. The fourth section had seven questions about “how often the respondents were bothered by the following problems in the last two weeks?” with the options - not at all, several days, more than half of the days, and nearly every day to measure anxiety among them. The final section was to measure sleep disturbances. This section contained nineteen structured questions about their overall sleep quality during the last month. Finally, these nineteen questions were grouped into seven components to calculate the sleep equally score on a four-point scale [15], [16], [17], [18]. After the collection of data, we analyzed them using Microsoft Excel (version 2016). We calculated the frequency and percentage of collected data and presented it in table format. However, the collected information using electronic platforms may not always be representative of the population.

## Ethics Statement

Committee for Advanced Studies at the Department of Pharmacy, University of Asia Pacific approved this study protocol (No. UAP/Pharm/2021/01004). We obtained electronic informed consent from all participants for this study. Also, we took informed consent from legal guardians in the case of minors who participated in the study.

## CRediT authorship contribution statement

**Md. Rabiul Islam:** Visualization, Data curation, Writing – original draft, Supervision. **Md. Ismail Tushar:** Data curation, Formal analysis, Writing – original draft. **Sanjida Jannath:** Data curation, Formal analysis, Writing – original draft. **Amena Ahmed Moona:** Visualization, Data curation, Writing – original draft, Supervision, Formal analysis. **Shahinur Akter:** Data curation, Formal analysis, Writing – original draft. **Sardar Mohammad Ashraful Islam:** Visualization, Data curation, Writing – original draft, Supervision.

## Declaration of Competing Interest

The authors do not have any conflict of interest to declare.

## Appendix Supplementary materials

Image, application 1

Image, application 2
