# Supplementary material for: Data set concerning the use of social networking sites and mental health problems among the young generation in Bangladesh
Source: Data Brief. 2021 Nov 19;39:107593. doi: 10.1016/j.dib.2021.107593 (PMC8627992; doi:10.1016/j.dib.2021.107593)
Supplement: Supplementary file 1 [file mmc1.docx]

A survey on the use of social networking sites and mental health of young generation in Bangladesh

**Please tick one box for each statement**

*Section 1: Demographic questions*

1. *Express your consent to participate in the research and processing of anonymous data for scientific purposes. Also, please confirm that you are a current used of social networking sites and your age is within 40 years.
   - Agree
2. *Age in years

- Below 18
- 18-25
- 26-40

1. *Sex

- Male
- Female

1. *Weight and height ratio/BMI score
   - Below 18.5 (underweight)
   - 18.5-25 (normal)
   - Above 25 (overweight)
2. *Marital status
   - Unmarried
   - Married
   - Others
3. *Education level
   - Primary
   - Secondary
   - Graduate/above
4. *Occupation
   - Service
   - Business
   - Student
   - Others
   - Unemployed
5. *Economic status
   - Low
   - Medium
   - High
6. *Smoking habit
   - Smoker
   - Nonsmoker
7. *Residence area
   - Urban
   - Rural

*Section 2: Questions related to the usage patterns of social networking sites*

1. *Which social media account do you use usually?

- Facebook
- Others

1. *Which device do you usually use to connect social media?

- Mobile phone
- Others

1. *Which type of internet connection do you use?
   - Broadband (Wi-Fi)
   - Mobile data
2. * How long have you been using a social media account?
   - Below 5 years
   - 5-10 years
   - More than 10 years
3. * How frequently do you post (upload status or add photos/videos) on social media?
   - Less than 1
   - 1-5
   - More than 5
4. * How much time do you spend daily in social media?
   - Below 1 hour
   - 1-3 hours
   - More than 3 hours
5. * When do you usually use social media?
   - At anytime
   - At specific time
6. * How many friends do you have on social media?
   - Below 500
   - 500-2000
   - Above 2000
7. *How many groups you are tagged in social media?
   - Less than 5
   - 5-10
   - More than 10
8. *What is your main purpose for using social media?

- Social communication
- Time passing
- Making new friends
- Others

1. * Do you believe social media is a good thing?
   - Yes
   - No
2. * When you see something in social media, do you instantly believe it?
   - Yes
   - No
3. * Have you ever experienced peer pressure due to social media?
   - Yes
   - No
4. * Does your emotion get influenced by other's posts?
   - Sometimes
   - Always
   - Not at all
5. * Have you ever compared yourself with other’s success or luxurious life?
   - Sometimes
   - Always
   - Not at all
6. * Do you think, your mental wellbeing would be better if you do not use social media?
   - Yes
   - No
7. *If answer is yes, are you trying to control that thing and trying to reduce the use of social media?
   - Yes
   - No

*Section 3: Psychometric assessment*

Indicate how often each of the statements below is descriptive of you.

**Loneliness Scale (UCLA-8)**

1. *In the past 30 days, I lack companionship.
   - Never (0)
   - Rarely (1)
   - Sometimes (2)
   - Often (3)
2. *In the past 30 days, there is no one I can turn to.
   - Never (0)
   - Rarely (1)
   - Sometimes (2)
   - Often (3)
3. *In the past 30 days, I feel left out.
   - Never (0)
   - Rarely (1)
   - Sometimes (2)
   - Often (3)
4. *In the last 30 days, I feel isolated from others.
   - Never (0)
   - Rarely (1)
   - Sometimes (2)
   - Often (3)
5. *In the last 30 days, I am unhappy being so withdrawn.
   - Never (0)
   - Rarely (1)
   - Sometimes (2)
   - Often (3)
6. *In the last 30 days, people are around me but not with me.
   - Never (0)
   - Rarely (1)
   - Sometimes (2)
   - Often (3)
7. *In the last 30 days, I am an outgoing person.
   - Never (0)
   - Rarely (1)
   - Sometimes (2)
   - Often (3)
8. *In the last 30 days, I can find companionship when I want it.
   - Never (0)
   - Rarely (1)
   - Sometimes (2)
   - Often (3)

**Patient Health Questionnaire-9 (PHQ-9)**

1. *In the last two weeks, little interest or pleasure in doing things.
   - Not at all (0)
   - Several days (1)
   - Half of days (2)
   - Nearly every day (3)
2. *In the last two weeks, feeling down, depressed or hopeless.
   - Not at all (0)
   - Several days (1)
   - Half of days (2)
   - Nearly every day (3)
3. *In the last two weeks, trouble falling or staying asleep, sleeping too much
   - Not at all (0)
   - Several days (1)
   - Half of days (2)
   - Nearly every day (3)
4. *In the last two weeks, feeling tired or having little energy.
   - Not at all (0)
   - Several days (1)
   - Half of days (2)
   - Nearly every day (3)
5. *In the last two weeks, poor appetite or over-eating.
   - Not at all (0)
   - Several days (1)
   - Half of days (2)
   - Nearly every day (3)
6. *In the last two weeks, feeling bad about yourself-or that you are a failure or have let yourself or your family down.
   - Not at all (0)
   - Several days (1)
   - Half of days (2)
   - Nearly every day (3)
7. *In the last two weeks, trouble concentrating on things, such as reading the newspaper or watching television.
   - Not at all (0)
   - Several days (1)
   - Half of days (2)
   - Nearly every day (3)
8. *In the last two weeks, moving or speaking so slowly or the opposite-moving around a lot more than usual.
   - Not at all (0)
   - Several days (1)
   - Half of days (2)
   - Nearly every day (3)
9. *In the last two weeks, thoughts that you would be better off dead, or of hurting yourself.
   - Not at all (0)
   - Several days (1)
   - Half of days (2)
   - Nearly every day (3)

**Generalized Anxiety Disorder Scale (GAD-7)**

1. *In the last two weeks, I am feeling nervous, anxious, or on edge.
   - Not at all (0)
   - Several days (1)
   - More than half of the days (2)
   - Nearly every day (3)
2. *In the last two weeks, I am not being able to stop or control worrying.
   - Not at all (0)
   - Several days (1)
   - More than half of the days (2)
   - Nearly every day (3)
3. *In the last two weeks, I am worrying too much about different things.
   - Not at all (0)
   - Several days (1)
   - More than half of the days (2)
   - Nearly every day (3)
4. *In the last two weeks, I feel trouble in relaxing.
   - Not at all (0)
   - Several days (1)
   - More than half of the days (2)
   - Nearly every day (3)
5. *In the last two weeks, I am being so restless that it's hard to sit still.
   - Not at all (0)
   - Several days (1)
   - More than half of the days (2)
   - Nearly every day (3)
6. *In the last two weeks, I becoming easily annoyed or irritable.
   - Not at all (0)
   - Several days (1)
   - More than half of the days (2)
   - Nearly every day (3)
7. *In the last two weeks, I am feeling afraid as if something awful might happen.
   - Not at all (0)
   - Several days (1)
   - More than half of the days (2)
   - Nearly every day (3)

**Pittsburgh Sleep Quality Index**

1. * During the past month, when have you usually gone to bed at night?
   - Before 10.00 PM
   - 10.01 PM to 12.00 AM
   - 12.01 AM to 2.00 AM
   - After 2.00 AM
2. * During the past month, how long (in minutes) has it take you to fall asleep each night?

- Less than 15 minutes
- 15-30 minutes
- 31-60 minutes
- More than 60 minutes

1. * During the past month, when have you usually gotten up in the morning?

- Before 5.00 AM
- 5.00 AM to 7.00 AM
- 7.01 AM to 9.00 AM
- After 9.00 AM

1. * During the past month, how many hours of actual sleep did you get at night?

- Less than 4 hours
- 4 to 6 hours
- 7 to 8 hours
- More than 8 hours

1. * During the past month, how many hours do you spend in bed?

- Less than 5 hours
- 5 to 7 hours
- 8 to 10 hours
- More than 10 hours

1. * During the past month, how many times, you cannot get to sleep within 30 minutes?

- Not during last month (0)
- Less than once a week (1)
- Once or twice a week (2)
- Three or more in week (3)

1. * During the past month, how many times, you wake up in the middle of the night or early morning?

- Not during last month (0)
- Less than once a week (1)
- Once or twice a week (2)
- Three or more in week (3)

1. * During the past month, how many times, you have to get up to use the bathroom?

- Not during last month (0)
- Less than once a week (1)
- Once or twice a week (2)
- Three or more in week (3)

1. * During the past month, how many times, you cannot breathe comfortably?

- Not during last month (0)
- Less than once a week (1)
- Once or twice a week (2)
- Three or more in week (3)

1. * During the past month, how many times, you cough or snore loudly?

- Not during last month (0)
- Less than once a week (1)
- Once or twice a week (2)
- Three or more in week (3)

1. * During the past month, how many times, you feel too cold?

- Not during last month (0)
- Less than once a week (1)
- Once or twice a week (2)
- Three or more in week (3)

1. * During the past month, how many times, you feel too hot?

- Not during last month (0)
- Less than once a week (1)
- Once or twice a week (2)
- Three or more in week (3)

1. * During the past month, how many times, you had bad dreams?

- Not during last month (0)
- Less than once a week (1)
- Once or twice a week (2)
- Three or more in week (3)

1. * During the past month, how many times, you have pain during sleep?

- Not during last month (0)
- Less than once a week (1)
- Once or twice a week (2)
- Three or more in week (3)

1. * During the past month, how many times, you have trouble in sleeping because of any other reason?

- Not during last month (0)
- Less than once a week (1)
- Once or twice a week (2)
- Three or more in week (3)

1. * During the past month, how often have you taken medicine to help you sleep?

- Not during last month (0)
- Less than once a week (1)
- Once or twice a week (2)
- Three or more in week (3)

1. * During the past month, how many times you did not sleep due to any program or other important case?

- Not during last month (0)
- Less than once a week (1)
- Once or twice a week (2)
- Three or more in week (3)

18. * During the past month, how much of a problem has it been for you to keep up enough enthusiasm to get things done?

- Not during last month (0)
- Less than once a week (1)
- Once or twice a week (2)
- Three or more in week (3)

19. * During the past month, how would you rate your sleep quality overall?

- Very good (0)
- Fairly good (1)
- Fairly bad (2)
- Very bad (3)

Any comments

……………………………………………………………………………………………………..

Thank you for completing this survey

*Mandatory Questions

Tip: The questionnaire includes skip Logic questions
